# Esophageal Anastomosis Medial to Preserved Azygos Vein in Esophageal Atresia with Tracheoesophageal Fistula: Restoration of Normal Mediastinal Anatomy

**Published:** 2012-10-01

**Authors:** Kumar Abdul Rashid, Madhukar Maletha, Tanvir Roshan Khan, Ashish Wakhlu, Jiledar Rawat, Shiv Narain Kureel

**Affiliations:** Department of Pediatric Surgery, King George Medical University, Lucknow, India

**Keywords:** Esophageal atresia, Tracheoesophageal fistula, Azygos vein preservation

## Abstract

Objective: We intended to prospectively study the technical feasibility and advantages of esophageal anastomosis medial to the preserved azygos vein in neonates diagnosed with esophageal atresia with tracheoesophageal fistula (EA/TEF). The results were compared to the cases where azygos vein was either not preserved, or the anastomosis was done lateral to the arch of preserved azygos vein.

Material and methods: A total of 134 patients with EA/TEF were admitted between January 2007 and July 2008 of which 116 underwent primary repair. Eleven patients with long gap esophageal atresia with or without tracheoesophageal fistula and 7 patients who expired before surgery were excluded. Patients were randomly divided in three groups comparable with respect to the gestational age, age at presentation, sex, birth weight, associated anomalies and the gap between the pouches after mobilization: Group A (azygos vein ligated and divided), Group B (azygos vein preserved with esophageal anastomosis lateral to the vein), and Group C azygos vein preserved with esophageal anastomosis medial to the vein). All the patients were operated by extra-pleural approach. The three groups were compared with respect to operative time and early postoperative complications like pneumonitis, anastomotic leaks and mortality. Odds ratio and Chi square test were used for the statistical analysis.

Results: Group A, B and C had 35, 43 and 38 patients respectively. No significant difference was observed in average operative time in the 3 groups. Though incidence of postoperative pneumonitis was higher in group A (28%) as compared to group B (13.95%) and group C (11.62%), it was not statistically significant (p > 0.005). Anastomotic leak occurred in 7 patients in group A (20%), 6 patients in group B (13.95%) and 4 patients (10.52%) in group C (p > 0.005). Group A had 3 major and 4 minor anastomotic leaks; group B had 2 major and 4 minor leaks and group C had 1 major and 3 minor leaks. There were10 deaths in the series- 5 in group A, 3 in group B and 2 in group C (p > 0.005). Patients with major anastomotic leaks in all 3 groups expired after re-exploration. The minor leaks were managed conservatively and all of them healed spontaneously. Severe pneumonitis and septicemia in patients having major associated anomalies also contributed to the mortality.

Conclusions: Although esophageal anastomosis medial to the preserved azygos vein restores the normal mediastinal anatomy without technical difficulty or increased operative time, the study could not prove a statistically significant advantage in terms of mortality and postoperative complications.

## INTRODUCTION

In standard technique for repair of esophageal atresia with tracheoesophageal fistula (EA/TEF), the azygos vein is ligated and divided before the mobilization of esophageal pouches [1-3]. Preservation of azygos vein has been found to maintain the mediastinal venous drainage and hemodynamic stability thereby improving wound healing and preventing postoperative complications like pneumonitis and anastomotic leaks by decreasing chest congestion and tissue edema [4, 5]. Although esophageal anastomosis can be performed lateral or medial to preserved azygos arch, yet it has been mostly done lateral to the azygos arch for the ease of the procedure. The present study is to evaluate the advantages, if any, of the esophageal anastomosis medial to the arch of the preserved azygos vein. The results are compared to the cases where azygos vein was ligated and divided or the anastomosis was done lateral to the arch of preserved azygos vein.

## MATERIAL AND METHODS

This prospective comparative study was conducted to analyze the technical feasibility and the advantages of esophageal anastomosis medial to the preserved azygos vein in neonates diagnosed with esophageal atresia with tracheoesophageal fistula. All patients with EA/TEF who underwent primary repair in the Department of Pediatric Surgery at King George Medical University, Lucknow between January 2007 and July 2008 are included in the study. The patients with long gap esophageal atresia with or without tracheoesophageal fistula, where the primary esophageal anastomosis was not possible, were excluded from the study. The patients were assigned to three groups by simple randomization: Group A in whom azygos vein was ligated and divided, Group B where the azygos vein was preserved and esophageal anastomosis done lateral to the vein, and Group C where azygos vein was preserved and esophageal anastomosis done medial to the vein. All the patients were operated by extra-pleural approach. The three groups were comparable with respect to the gestational age, age at presentation, sex, birth weight, associated anomalies and the gap between the pouches after mobilization. The parameters used to compare the results in the three groups were the operative time and early postoperative complications like pneumonitis, anastomotic leaks and mortality.

Technique:

Through right posterolateral thoracotomy, posterior mediastinum is reached by gently sweeping the parietal pleura off the endothoracic fascia with the help of wet pledgets. Azygos vein is seen crossing from right to left. A nick is made in the endothoracic fascia just below the azygos arch. A blunt right angled forceps is passed beneath the azygos arch and a tunnel is made by stripping off the endothoracic fascia above it (Fig. 1).

**Figure:**
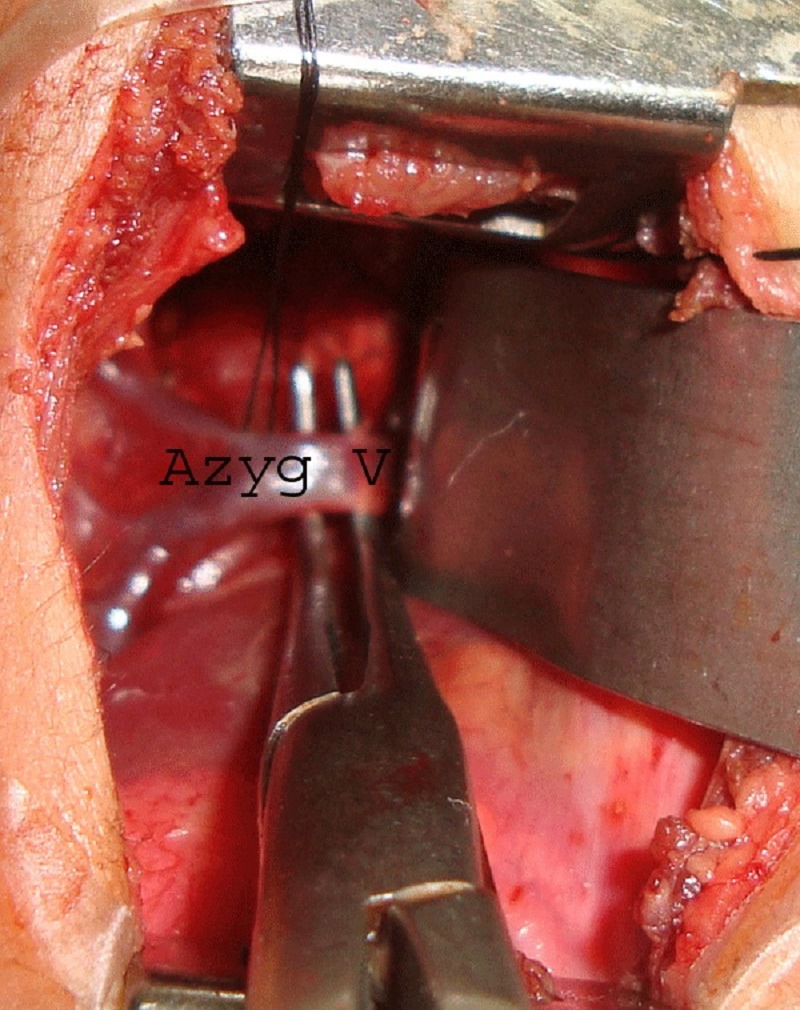
Figure 1: A tunnel is being created medial to the preserved arch of azygos vein

If the lower esophageal pouch ends in a fistula below the azygos arch, it is identified and the fistula is divided and closed either by interrupted non-absorbable sutures or by transfixation. The lower divided end of esophagus is passed through the tunnel beneath the azygos arch and is anastomosed end to end with the upper esophageal pouch after its adequate mobilization. In case the lower esophageal pouch is ending in a fistula with the trachea at a higher level, it can be hooked above the azygos arch with the help of a gentle downward traction on the azygos arch (Fig. 2). The anastomosed neoesophagus lies medial to the azygos vein as in normal subjects (Fig. 3). 

**Figure:**
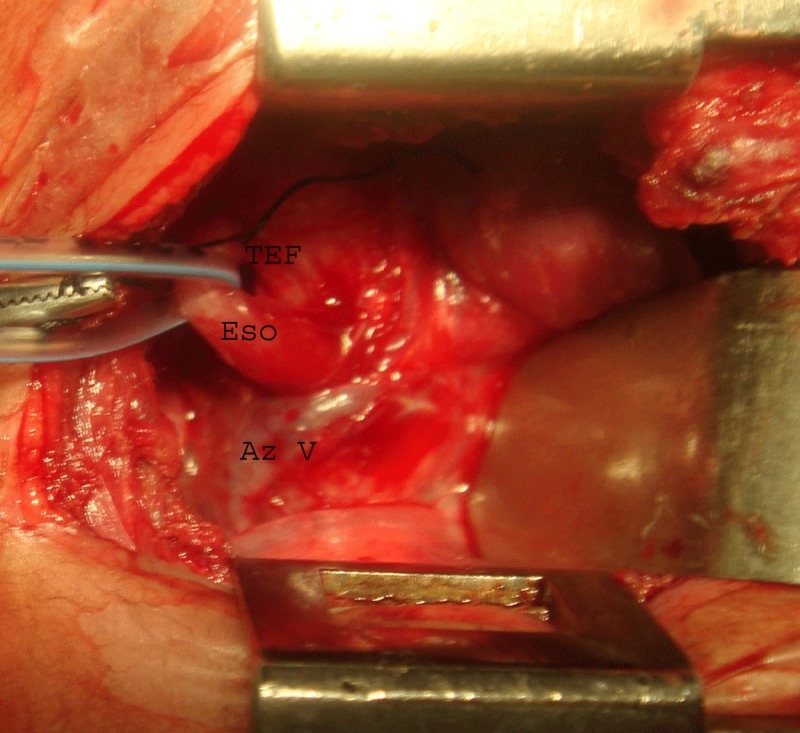
Figure 2: Lower esophageal pouch hooked superior to azygos arch with a transfixation suture in place at the fistula site.

**Figure:**
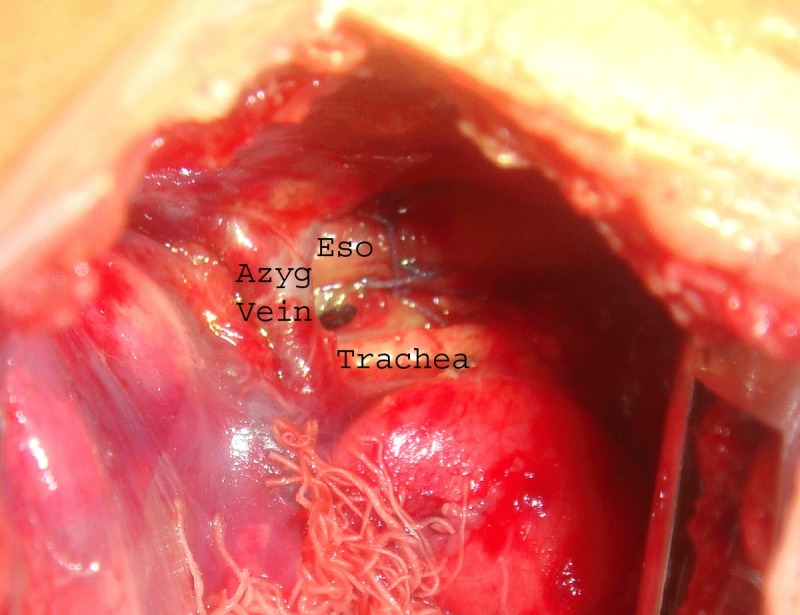
Fig. 3: Anastomosis completed with the neo-esophagus lying medial to the preserved azygos vein

A trans-anastomotic 6 Fr feeding tube is passed during anastomosis for early postoperative enteral feeding. A retropleural drain may or may not be placed according to the operating surgeon's preference. Feeding through trans-anastomotic tube is started after 24 hours of surgery in all patients. All the patients receive same pre and postoperative treatment. Oral feeds are started following a water soluble contrast esophagoraphy after 6th postoperative day. In patients where a retropleural drain is placed minor leaks are identified by appearance of frothy saliva in the drain without deterioration of general condition of the patient while the major leaks are suspected by profuse drainage through the drain with deterioration in general condition of the patients in the form of increasing respiratory distress and septicemia.

## RESULTS

The comparison between three groups in terms of sex, birth weight, age at presentation, prematurity, associated anomalies and gap length after adequate mobilization is presented in Table 1. Comparison of results in terms of operative time, early postoperative complications and mortality are presented in Table 2. No significant difference was observed in average operative time. Postoperative pneumonitis was higher for group A as compared to groups B and C, but this was not statistically significant (p>0.005). Anastomotic leak occurred in 17 patients in the series. There was no statistical difference between the 3 groups (p > 0.005). There were10 deaths in the series- 5 in group A, 3 in group B and 2 in group C (p > 0.005). All patients with major anastomotic leaks in group A and group B (3 and 2 respectively) expired after re-exploration with esophagostomy and feeding gastrostomy. One patient with major leak in group C also died after re-exploration and esophageal re-anastomosis. The minor leaks were managed conservatively and all of them healed spontaneously. This was confirmed by contrast esophagography and patients were allowed orally only after complete healing at anastomotic site was documented. Severe pneumonitis and septicemia in patients having major associated anomalies also contributed to the mortality.

**Figure:**
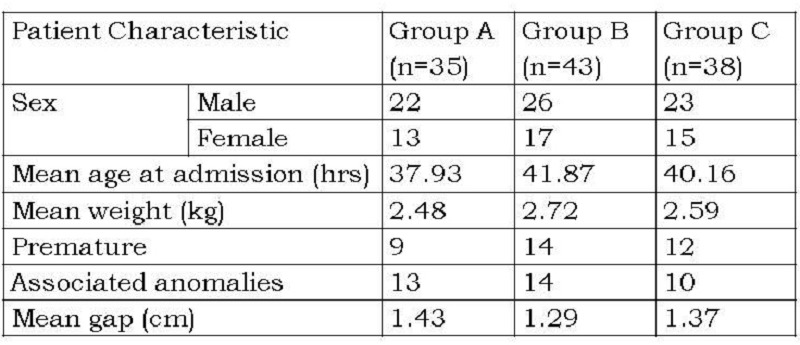
Table 1 Comparison of patient characteristics in the three groups

**Figure:**
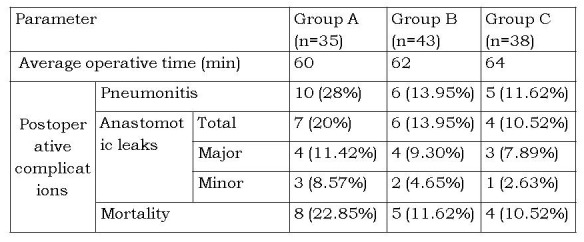
Table 2 Comparison in terms of operative time and postoperative complications

## DISCUSSION

The evolution from a congenital abnormality incompatible with life to a condition with a survival rate crossing well over 90% is a remarkable achievement in cases of esophageal atresia (EA) with tracheoesophageal fistula (TEF). Since 1888 when Charles Steele [6] first attempted surgical repair in a patient of EA without TEF, several technical modifications have been made to improve the surgical outcome of these patients and to reduce mortality and complication rates. At present, the standard technique is to ligate and divide the azygos vein early in the operation. This is supposed to provide an easy access to the atretic esophageal ends and the fistula and also to make the anastomosis comfortable. The azygos system reflects two primary objectives of venous system design; one is to provide multiple routes for blood to return and another to take the least complicated course from the tissue source to the major vein of passage. At the beginning of the azygos arch, the right superior intercostal vein joins it, having received the 2nd, 3rd, and 4th posterior intercostal veins. At the level of the 4th thoracic vertebra, the azygos vein arches above the hilar structures of the right lung to enter the superior vena cava, with esophagus medial to it in the posterior mediastinum [7]. Preservation of azygos vein has been found to maintain the mediastinal venous drainage, thereby decreasing postoperative chest congestion and tissue edema [4, 5]. This in turn would prevent postoperative complications like pneumonitis and anastomotic leaks by improving wound healing [4, 5]. Although esophageal anastomosis can be performed lateral or medial to preserved azygos arch, yet it has been mostly done lateral to the azygos arch for the ease of the procedure. We did not find anastomosis medial to preserved arch of azygos vein technically difficult nor was the operative time significantly increased. Consistent with the previous studies [4,5], the complications and mortality in azygos preserving groups (groups B and C) although lower than in group A where the azygos was ligated (Table 2), are not significantly different. We prefer to anastomose the esophageal ends medial to the preserved azygos arch as it restores the normal anatomical relation of the mediastinal structures (Fig. 3) and provides the shortest route for the neo-esophagus. 

## CONCLUSION

The esophageal anastomosis medial to the preserved azygos vein in EA/TEF does not provide a statistically significant advantage in terms of mortality and postoperative complications. We still propose to restore the normal mediastinal anatomy by this technique as it is not technically difficult and does not increase the operative time.

## Footnotes

**Source of Support:** Nil

**Conflict of Interest:** None declared
